# Use of Digital Health and Digital Therapeutics to Treat SUD in Criminal Justice Settings: a Review

**DOI:** 10.1007/s40429-023-00523-1

**Published:** 2023-12-27

**Authors:** Ginnie Sawyer-Morris, Judith A. Wilde, Todd Molfenter, Faye Taxman

**Affiliations:** 1 Addiction Policy Forum, 4701 Sangamore Rd, Ste 100N—1173, Bethesda, MD 20816, USA; 2 Schar School of Policy and Government, George Mason University, 3351 Fairfax Drive, Arlington, VA 22201, USA; 3 Albuquerque, USA; 4 Center for Health Enhancement Studies, University of Wisconsin-Madison, 1513 University Avenue, Madison, WI 53706, USA

**Keywords:** Digital therapeutics, Digital health, Software as a medical device, Substance use disorder, Opioid use disorder, Criminal justice settings

## Abstract

**Purpose of Review:**

The purpose of this review is to investigate the use of digital health technologies and/or digital therapeutics (DTx) products in the treatment of substance use disorders (SUDs) in the general population and among criminal justice–involved individuals.

**Recent Findings:**

Despite an expanding evidence base, only three SUD DTxs have received federal regulatory approval. Across studies, DTx products have proven successful in engaging patients in SUD treatment and reducing healthcare costs and resource utilizations. Findings for emerging SUD DTx products show similar results. Still, there is a paucity of evidence regarding the use of digital health technologies and/or DTx among criminal justice populations.

**Summary:**

DTxs have proven effective for treating multiple SUD types (e.g., nicotine and opioids) among the general population. DTx shows similar promise among justice-involved populations, but additional efficacy and implementation research is needed to address barriers such as cost, cultural resistance, and infrastructure.

## Introduction

**Fig. 1:**
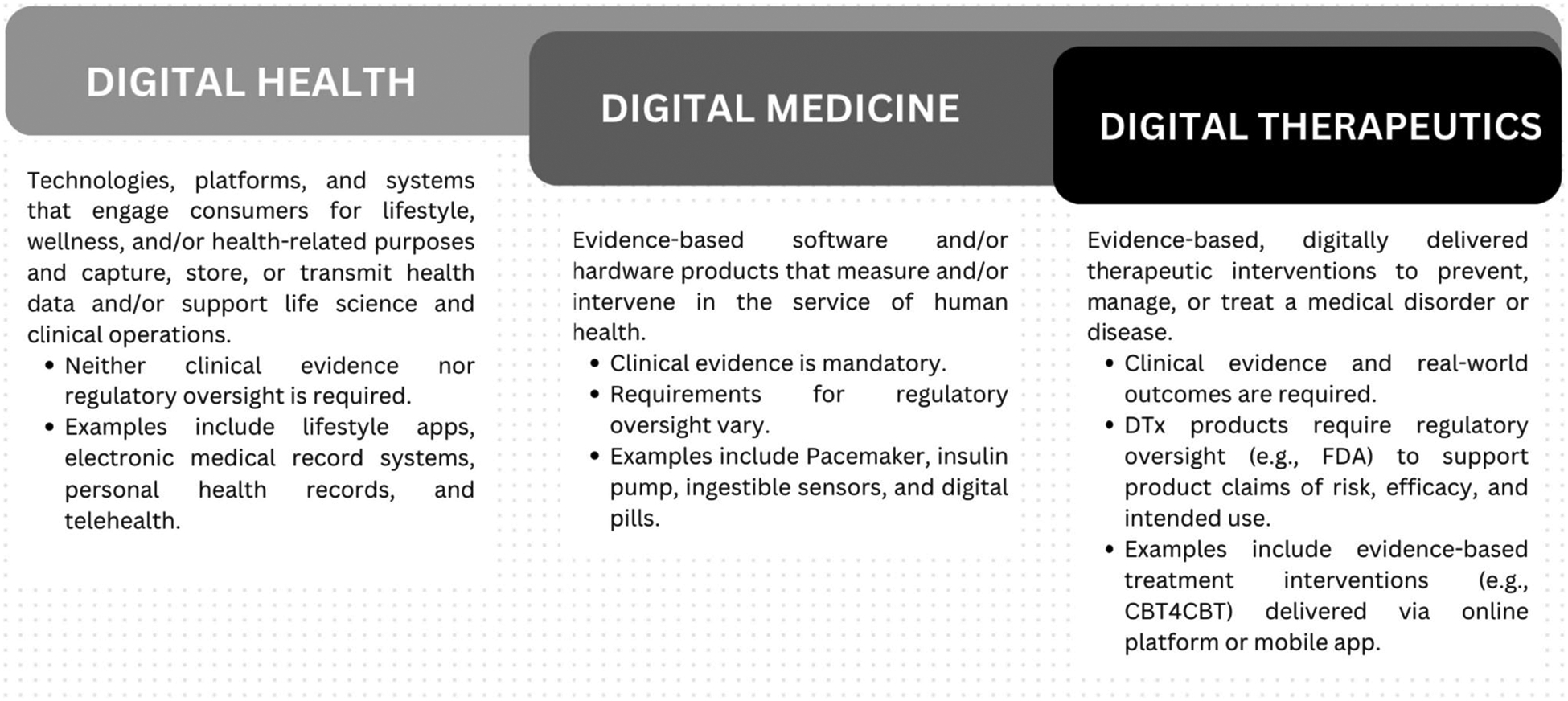
Depiction of differences among digital health, digital medicine, and DTx products [4, 24]

The move toward digital healthcare nearly 30 years ago has empowered patients to participate in their own care. The digital health umbrella (see Fig. 1) encompasses a growing range of technologies including digital health products that encourage clients to maintain healthy lifestyles (e.g., mobile apps Reframe, HereNOW Connect, and social media platforms [1, 2]), digital medicine (e.g., ingestible sensors, digital pills), software as a medical device (SaMD), and digital therapeutics (DTx; digitally delivered interventions [3–5]).

DTx is a growing subfield of digital health focusing on conditions that can be treated, managed, or prevented through digital interventions (e.g., computer-based training for cognitive behavioral therapy) [5–7]. Both DTx and SaMD represent software used for medical purposes, with SaMD intended to diagnose, mitigate, treat, and prevent disease [8] and DTx limited to treatment based on clinical evidence of a potential disease or disorder although it “does not act in the human body through pharmacological, immunological or metabolic ways” [4]. As such, DTxs are used to treat a variety of conditions including long-term management of diabetes and prediabetes [9], improved motor outcomes in Parkinson’s disease [10], greater adherence to medications for hypertension and cardiovascular disease [11], mental health improvement with cognitive rehabilitation [12], improved obstetrical outcomes (e.g., preterm labor) [13], improved early diagnosis/intervention for children with autism [14], and increasing abstinence and treatment retention for individuals with substance use disorders [15, 16•, 17]. Evidence from clinical trials and real-world evaluations of DTx and digital health suggests that clients view these treatments as convenient, efficient, comfortable, and private, sometimes rating digital health experiences as more effective than usual care [18, 19]. There also is some evidence that adherence to DTx is greater than to traditional treatment with medication [5].

While advantageous due to their scalability, accessibility, cost-effectiveness, and cultural adaptability, DTxs require clearance from a regulatory body (e.g., Food and Drug Administration (FDA)). FDA approval can be a long, expensive, and arduous path for medications—an approval process requiring demonstrated efficacy in both clinical and real-world settings [20–22] as well as identifying any risk factors [8]. The FDA regulates DTxs that meet the definition of SaMD primarily through two processes: de novo and 510(k). The former is used for low-risk medical devices and requires “reasonable assurance of safety and effectiveness for the intended use”; the latter is used when developers are able to show that their new DTx is substantially similar to one or more DTxs that already are available [8]. Data for FDA approval come from both exploratory and confirmatory studies [5].

Several companies have developed, or are developing, DTx to treat substance use disorder (SUD) but the market remains small. Currently, only two SUD DTx developers have received regulatory approval from the FDA: Pear Therapeutics (reSET and reSET-O [22]) and DynamiCare (DynamiCare Health [23]). Nonetheless, it is estimated that by 2025, the global DTx field will be valued at $7 billion to nearly $11 billion [4, 24, 25].

Despite the projected growth, DTx remains underused and understudied [26, 27], and little evidence exists on the effectiveness of digital health technologies and DTx with justice-involved populations (e.g., drug courts, probation, jail/prison). This is particularly troubling given that justice involvement and SUD often go hand-in-hand [28]. At least 65% of those within the US prison system have had an SUD [29], and drug overdose is the leading cause of death in the weeks following parole or probation [29–31].

### What Digital Health and DTx Are Available for Criminal Justice Populations?

Individuals with SUD and justice involvement have high treatment needs yet are among the least represented populations in digital health and DTx research and treatment efforts. Recent data from the Bureau of Justice estimate that 85% of the prison population either have an active SUD or were incarcerated for a crime involving drugs or drug use [30]. Among incarcerated individuals with SUD, only 33% of state prisoners and 46% of federal prisoners receive treatment [31].

Justice-involved individuals with SUD face barriers to treatment both within and outside carceral contexts, and while digital health has been cited as a potential solution for engaging hard-to-reach populations (e.g., rural communities, justice-involved populations) in SUD treatment, justice-involved populations remain underrepresented in digital health literature [32••]. Furthermore, individuals accessing treatment while incarcerated face challenges maintaining their recovery upon reentry. These include a lack of social and family support, financial insecurity, loss of income derived from drug sales, inadequate housing [33•], and stigma [20]. In outpatient contexts, justice-involved individuals who are referred to treatment consistently report more severe SUD symptoms and greater resistance to treatment compared to individuals who self-refer [34, 35].

#### Digital Health Adoption in Correctional Settings

Correctional facilities were among the first settings evaluated for digital health adoption. In 1994, the US Departments of Justice and Defense established a 3-year digital health demonstration project in correctional settings to assess whether a correctional digital health network would improve access to specialty healthcare, lower security risks, and decrease healthcare costs [36]. Results demonstrate that, depending on the type and nature of the institution, digital health modalities (e.g., videoconferencing) could improve inmate access to specialty healthcare while reducing costs [37••]. Included among the project’s recommendations for facilities considering digital health adoption was a cost–benefit analysis comparing the cost of Medicare and technological requirements with the institution’s individual- and organizational-level resources (e.g., proximity to major healthcare providers, budget) [38].

By 2001, half of state correctional institutions and 39% of federal institutions employed digital health services (e.g., telemedicine) to provide healthcare to incarcerated individuals—this typically involved specialty care [39]. While criminal justice settings were early adopters of digital health, the type and extent to which digital health services were implemented varied across facilities. More than 20 years later, and despite decreases in the cost of digital health resources, the mode and application of services in correctional settings remain variable [40, 41•]. Key barriers include, but are not limited to, costs, security risks, coordination of services, organizational- and individual-level factors (e.g., attitudes of inmates and correctional officers toward technology/change), and legal and regulatory procedures [42•].

Since 2019, Justice Community Opioid Innovation Network (JCOIN) researchers in Massachusetts have been studying digital health tools and platforms that provide opioid use disorder (OUD) treatments for justice-involved individuals. The purpose of the research is to “address gaps in OUD treatment and related services in a wide range of criminal justice settings, including jails, drug courts, problem-solving courts, policing and diversion, reentry, and probation and parole” [43].

The aims of this review are to (1) investigate the design and implementation of DTx for the treatment of SUD, (2) identify DTxs that have been used in justice settings, and (3) examine the implementation challenges for DTx in justice settings.

## Methods

The following definitions [3, 22] distinguish DTx from other forms of digital health.

*Digital health.* Technologies, platforms, and systems that engage consumers for lifestyle, wellness, and/or health-related purposes and capture, store, or transmit health data and/or support life science and clinical operations. Digital health technologies require neither clinical evidence nor regulatory oversight. Examples include lifestyle apps and electronic medical record systems.
*Digital Medicine.* Evidence-based software/hardware products measuring/intervening in the service of health. Clinical evidence is mandatory; requirements for regulatory oversight vary. Examples include pacemakers, insulin pumps, and digital pills.
*DTx.* Evidence-based therapeutic interventions preventing, managing, or treating medical disorders or diseases. Clinical evidence and real-world outcomes are required for DTx products which must be reviewed and cleared or certified by regulatory bodies (e.g., FDA) to support claims of risk, efficacy, and intended use. Examples include medically assisted SUD treatment augmented with digital delivery of evidence-based behavioral interventions such as cognitive behavioral therapy (CBT).

These definitions focused the search on existing literature with evidence regarding DTx for SUD treatment within and outside criminal justice settings. The search for peer-reviewed studies, especially after 2017, involved multiple databases including PubMed, Research Gate, Scopus, Google Scholar, and Ovid. More than 100 articles were identified; those most relevant to DTx or digital health, and providing the greatest depth of information, were maintained. A small number of older articles are included because either they are seminal to the field or more recent research was not identified.

**Fig. 2:**
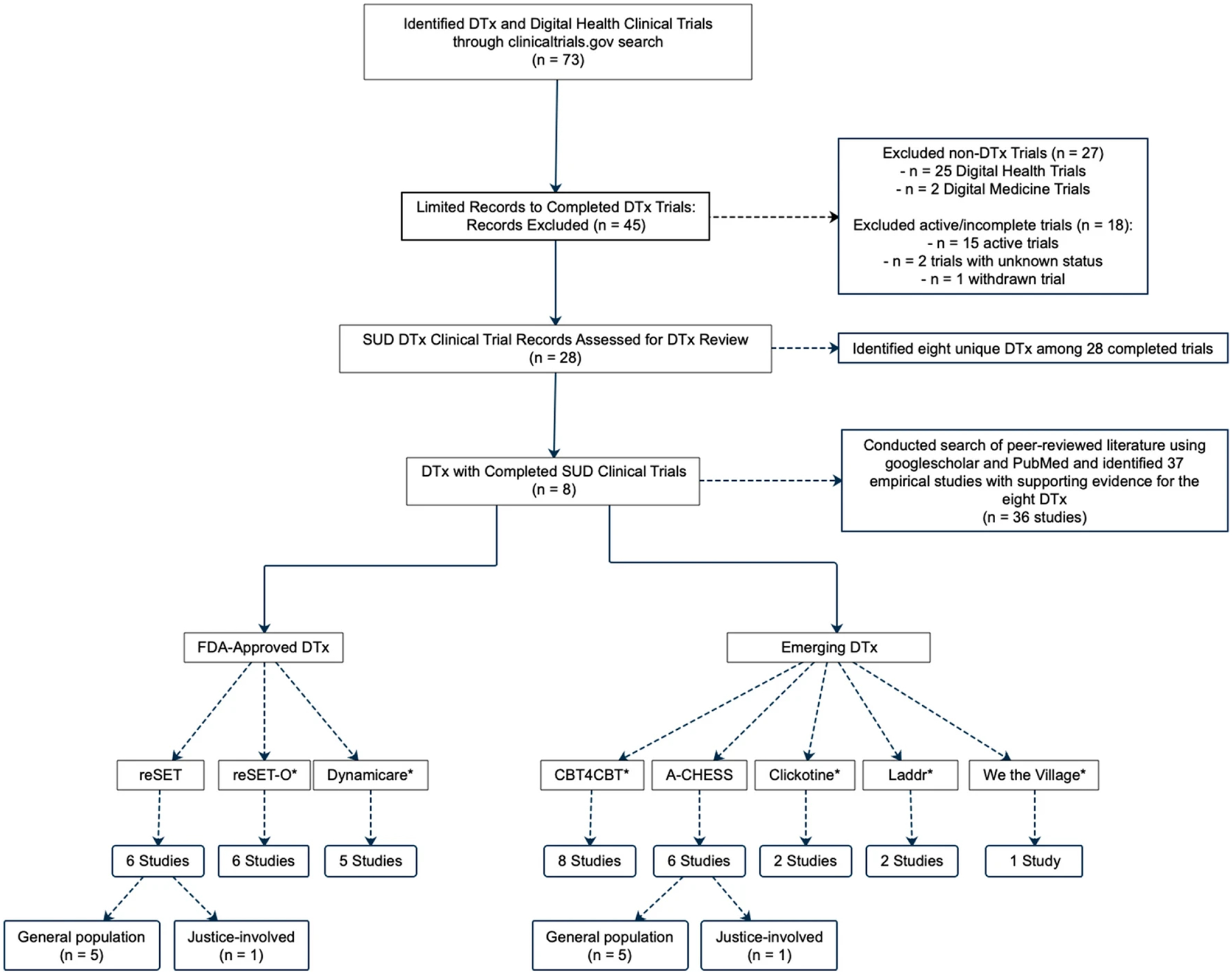
Decision tree for selection of the DTx in this review. *These DTxs have been studied only in the general population, not with justice-involved individuals

A search of clinicaltrials.gov used the terms “digital therapeutics,” “digital,” “mobile,” “phone,” “tele,” “alcohol,” “substance,” “tobacco,” “opioid,” “cocaine,” “stimulant,” and “drug.” Currently, clinicaltrials.gov does not distinguish between a DTx trial and a digital health trial. Figure 2 illustrates the manual review process that identified eight DTxs from among the original pool of 73 clinical trials. Other peer-reviewed literature was used to provide support around both the purpose and successes of both DTx and digital health.

Multiple searches were conducted using various combinations of term(s); findings are limited to research studies with comparison groups or experimental studies (see Table 1).

## Narrative Review

The terms “digital health,” “digital medicine,” and “DTx” have been used interchangeably in publications on digital health for SUD [3]. We provide an update on recent SUD DTx research, including digital health in criminal justice settings, and to distinguish between DTx and other digital health treatments.

### What DTxs Are Available for SUD Treatment?

Studies indicate that DTx for SUD can produce beneficial treatment effects (e.g., abstinence) for individuals with a variety of SUD types including nicotine/tobacco, alcohol, cocaine, cannabis, and opioids [44]. The DTxs discussed herein have FDA approval or have demonstrated promise through randomized clinical trials (RCTs).

#### FDA-Approved DTx for SUD

Although DTxs were first explored nearly 30 years ago [24], only two have FDA approval to treat multiple types of SUD, and a third has approval to treat nicotine addiction.

##### reSET

In 2017, the FDA authorized reSET, a mobile adaptation (app) of the web-based therapeutic education system (TES) and the first prescription DTx for treating patients with cocaine, cannabis, and/or stimulant use disorders [22]. reSET delivers self-directed and interactive CBT grounded in the community reinforcement approach (CRA) to enhance behavior change [45, 46]. Studies support the efficacy of reSET for improving treatment outcomes including abstinence in clinical [22, 45–49] and real-world settings [50•]. In addition, TES and standard treatment were equally effective in reducing criminality, relapse to drug use, and HIV risk behavior among substance-using offenders in prison contexts [46]. Recent studies indicate that using reSET reduces healthcare resource utilization and lowers per-patient healthcare costs by $3591 over a 6-month period [15]. Although reSET has a strong evidence base, more research is needed to explore best practices for implementing reSET across additional populations, treatment settings, and SUD types.

##### reSET-O

In 2018, the FDA authorized reSET-O, a mobile app with an 84-day DTx for OUD. reSET-O serves as an adjunct to outpatient treatment by providing CBT along with transmucosal buprenorphine and contingency management for patients 18 years or older who are under the supervision of a clinician. A feasibility study demonstrated that reSET-O is well accepted by both patients and clinicians [51]. A growing body of literature testing the effectiveness of reSET-O in real-world settings [52–56] shows it is associated with clinically significant treatment outcomes including abstinence from illicit opioids and retention in treatment [22, 52]. Studies from one research team also demonstrate significant and durable reductions in emergency department and inpatient utilization (including readmissions) [53], reduced net costs for third-party payers [54] and healthcare organizations [55], and increased clinician services [56]. These trends were observed across 6-, 9-, and 12-month post-treatment periods [53, 56]. Another study found that doubling the dosage (24 weeks instead of 12 weeks) increased retention in treatment and reduced hospital encounters [17].

The greatest cost benefits were among patients with Medicaid [55]. In their economic modeling study of a 1-million-member healthcare plan, Wang et al. [57] observed similar cost-effectiveness and potential cost-savings.

While reSET-O is supported by peer-reviewed literature with real-world evidence, its RCT evidence base still is emerging. Two reSET-O RCTs are registered on clinicaltrials.gov; one is estimated to be completed in September 2023 [58] and the other in March 2024 [59], but the latter has yet to begin recruitment. No results have been published for either trial. Additional RCTs with longitudinal outcomes (12–24-month follow-up) evaluating the clinical efficacy of reSET-O and its impact in preventing overdose are needed.

##### DynamiCare Health

In 2022, DynamiCare Health received FDA Breakthrough Device Designation for the treatment of smoking during pregnancy. DynamiCare Health is an evidence-based digital care program (contingency management plus recovery coaching) delivered through a smartphone app. The program offers up to $100/month for negative drug tests and appointment attendance. Subscribers also have access to a recovery coach by weekday texts and weekly phone and video. If needed, the coach will help set achievable goals related to recovery. Additional app features include video monitoring for alcohol abstinence, a log of substance use screening results, a Bluetooth-enabled breathalyzer, saliva drug testing, a “certified guide,” and appointment monitoring and reminders [23].

Several studies have examined DynamiCare’s contingency management software. Adults with alcohol use disorder (AUD) who used DynamiCare to supplement “usual care” treatment have greater success maintaining their sobriety than those who received usual care only [60], as did those with OUD [61–63]. Pregnant smokers had higher quit rates with financial incentives for actually practicing “best practices” [64]. An efficacy trial for SUD patients (NCT04162132) was completed in 2019; no results or studies have been published [65]. A 2022 clinical trial assessing contingency management apps’ effectiveness in treating multiple SUD types among older adults, including DynamiCare, has not begun participant recruitment.

Both reSET and reSET-O have demonstrated improved outcomes for SUD and OUD, and reSET-O is cost-effective, showing potential cost-savings. However, only one identified study used reSET in criminal justice settings [see 46]. Further implementation trials are underway. While the evidence base for DynamiCare Health grows, it currently is approved only for nicotine but has been studied for OUD [see 66]. More efficacy trials are needed to determine the effectiveness of all three DTx for different populations and SUD types.

#### Emerging DTx for SUD Treatment

We identified emerging research on DTx for SUD on clinicaltrials.gov using multiple search terms including types of digital support and SUD—alone and in various combinations. The SUD DTx interventions reported herein are limited to the five with completed trials (see Table 1), but none currently is FDA-approved.

##### Computer-Based Training for Cognitive Behavior Therapy (CBT4CBT)

CBT4CBT is a web-based seven-module DTx for SUDs based on a NIDA-published CBT intervention compendium [6]. Available in English and Spanish, CBT4CBT focuses on cognitive and behavioral skills to address substance use. Participants can complete the self-guided program on their own. CBT4CBT has a strong evidence base, including its effectiveness as an adjunct to standard outpatient treatment [67••], and is one of the most widely used digital interventions in the SUD DTx space. CBT4CBT has been validated for:

- Multiple SUD types including AUD [68] and cocaine [69];
- Multiple populations including women in residential treatment [70•] and Spanish speakers [71, 72••];
- Clinical [67••] and real-world settings [73•].

Follow-up studies of clinical trial data demonstrate CBT4CBT’s durable effects 6 months post treatment and as an augmentation to multiple types of medications for opioid use disorders (MOUD) including buprenorphine [73•] and methadone [69]. Individuals with higher risk may demonstrate poorer treatment outcomes in terms of substance use and lower rates of task completion [11]. However, participants generally report positive treatment outcomes including an increase in coping skills related to substance use recovery (e.g., coping with craving) [74].

##### A-CHESS

A-CHESS supports the treatment and recovery process by reducing the incidence and severity of relapse and increasing adherence to SUD treatment [75]. The A-CHESS platform (smartphone apps for patients 13 years and older, providers, and families) delivers recovery-oriented motivational content, social connections, and 24/7 support. Clinical trials demonstrate that A-CHESS reduces the risk of relapse, decreases substance use, and increases social support [76]. The app’s adaptive and extensive menu of services provides immediate support across multiple populations (e.g., Latinx [76], impoverished rural women [77]) and SUD types (e.g., AUD [78] and OUD [79]).

A secondary analysis of the A-CHESS RCT found that the effect of A-CHESS on risky drinking days was mediated by outpatient treatment [80]. Another study examined the feasibility of implementing the A-CHESS Connections app, offering CBT4CBT, peer support services, and one-on-one meetings with a social worker, among populations with both SUD and justice involvement. For clients with and without justice involvement, protective factors increased with app engagement; justice-involved clients preferred time spent with the social worker over other app services. In addition, those who seek assistance on their own, rather than being referred by a justice-related agency, are more likely to benefit [34].

##### Clickotine

Clickotine is a smartphone app designed and engineered to deliver components of an empirically supported smoking cessation intervention and essential features of the US Clinical Practice Guideline (USCPG; e.g., advise and encourage to quit, enhance motivation). As USCPG recommends, Clickotine uses an adaptive, proprietary technology platform, Clickometrics, to enhance engagement by personalizing the intervention to the client [81]. An initial trial reported that access and adherence to smoking cessation medications increased during an 8-week, single-arm clinical trial [82]. A second trial was registered in 2022 but was withdrawn citing a change in Click Therapeutics’ priorities and belief that the original trial (NCT04857515) was sufficient [83].

##### We The Village

We The Village is an online community forum and web-based course integrating Community Reinforcement and Family Training (CRAFT), an evidence-based intervention for concerned significant others (CSOs) of alcohol-dependent individuals (ADI). The program engages treatment-refusing patients and improves CSO functioning [84].

To date, there have been four RCTs of CRAFT-based treatment for CSOs of ADI (three in the USA, one international). The most recent RCT developed and refined digital delivery of two modified CRAFT interventions, CRAFT-A (group protocol) and CRAFT-C (protocol with live coaching), to assess their effectiveness compared to the business-as-usual model of a peer support forum (PEER). The goal was to deliver an innovative, scalable, and effective evidence-based behavioral intervention with consumer technology to an existing and growing online audience. The trial was completed in June 2022 but has not yet published findings. While previous CRAFT trials have demonstrated the intervention’s efficacy in engaging ADIs in treatment, research on digital delivery is still emerging.

##### Laddr^®^

Laddr^®^ is an evidence-based mobile platform integrating therapeutic processes to address a wide range of behavioral challenges [85]. Laddr^®^ is effective with multiple populations including Spanish speakers in Colombia with problematic alcohol use [86, 87]. To date, four clinical trials have been conducted: three used single-group assignment and one used non-randomized parallel assignment. The trials focused on self-regulation with applications to binge-eating, overweight, and smoking.

While research is ongoing, the current evidence base on the efficacy and effectiveness of Laddr^®^ for SUD treatment is limited; more research is needed. A randomized clinical trial (NCT05648786) to evaluate the effectiveness of Laddr^®^ among individuals with different SUD types is currently in the recruitment stages; its expected completion is the end of 2023 [87].

Across these five emerging DTxs, findings demonstrate that DTx technologies result in increased abstinence across multiple substances including opioids, cocaine, alcohol, and nicotine, as well as increased retention in treatment. All five support clients, while two support families and loved ones. All use some type of mobile or web-based platform, and four provide as-needed professional support. The evidence for Clickotine, We The Village, and Laddr^®^ is somewhat weak, and clinical trials for the two have not yet been reported.

Overall, these eight reviewed DTxs involve multiple populations, including women, pregnant women, Spanish speakers, Latinx, and older adults. Only A-CHESS has been studied with individuals with justice involvement [see 34]; none have been used within carceral settings. Given the large number of justice-involved individuals in the USA, it is essential that this lack of research be remedied.

#### Challenges to Implementing DTx for SUD in Criminal Justice Settings

Healthcare services delivered in correctional settings vary from healthcare provided by correctional employees to services delivered through community-based systems or by contracted health professionals [42•]. While DTxs represent a potential cost-cutting solution, research on the use and implementation of DTx for SUD in correctional settings is limited. Most available research focuses on psychological outcomes and mental health, and only a small number of digital health interventions for SUD treatment exist (see [32••, 35] for reviews of this literature). Of these, three meet the qualifications for DTx designation: MAPIT [88, 89••, 90, 91], TES for offenders with SUD [92••, 93••], and A-CHESS for drug court participants [94].

Promising partnerships and projects are being funded at the intersection of criminal justice and DTx. In 2022, Pear Therapeutics partnered with the South Carolina Department of Corrections to provide reSET and reSET-O to incarcerated women [95]. This program was successfully rolled out prior to Pear Therapeutics’ filing for chapter 11 in April 2023, an outcome that experts and competitors alike are attributing to a lack of market acceptance and too high an initial valuation without enough runway to drive revenue [96, 97]. Prior to the filing, Pear Therapeutics was working with eight additional states to provide similar services to incarcerated individuals [98]. Also in 2023, Florida approved Medicaid payments for reSet and reSet-O [99]. Despite Pear Therapeutics’ filing, this Medicaid approval of DTx in a large US state marks important progress for the field as it moves toward reimbursement where no such option previously existed [100].

While DTx partnerships are only beginning in criminal justice settings, the digital health (e.g., telemedicine) footprint is much more established. For example, New York City Health and Hospitals has been collaborating with Cisco since 2017 to provide access to specialty healthcare in 12 municipal jails. Cisco’s digital health technology (i.e., telemedicine) allows administrators to coordinate virtual visits with healthcare practitioners for the jail system’s residents. This partnership has saved transportation costs while providing incarcerated individuals with access to specialty care (e.g., SUD, behavioral health). This has the added benefits of being quicker, more personalized, and flexible as providers are able to deliver care on their own schedules [101].

Texas, which has the nation’s largest incarcerated population (more than 150,000), also utilizes digital health technology. Through a partnership with the University of Texas Medical Branch, Texas correctional facilities provide telemedicine to treat 80% of the incarcerated population [98]. Given the precedents set by the broader digital health field, justice-involved contexts represent a promising arena for the implementation of DTx at scale. However, the DTx field is young and more research (e.g., population-specific effectiveness research) is needed to evaluate the implementation and effect of emerging programs in criminal justice settings.

##### Intersection of Privacy and Distrust

DTx and digital health interventions require sensitive data collection, yet often researchers cannot fully guarantee data anonymity or privacy. Thus individuals may have data-related concerns about legal, social, and economic harms that can contribute to the challenge of participant acceptance of digital health, particularly in SUD interventions with justice-involved populations [102•]. Issues related to data privacy and the potential for healthcare distrust among incarcerated individuals exacerbate existing barriers to SUD treatment both within and outside carceral contexts. Researchers working in this domain should apply intersectional, ethical frameworks to the design and adaptation of digital interventions. Engaging communities and representative organizations in ongoing and meaningful consultations will mitigate potential harms and address concerns of this population [103].

##### Cultural Representation

While not a DTx study with justice-involved populations, Campbell et al. [104] did conduct an acceptability and feasibility study of TES among American Indian and Alaskan Native (AI/AN) populations. Results provide helpful insights on tailoring interventions to specific populations in a culturally appropriate fashion. Based on participants’ feedback, they recommend moderate adaptations related to delivery and presentation (e.g., casting an AI/AN actor to record audio and video components in a Native voice, use of stories rather than “academic” presentations). These adaptations demonstrate how DTx interventions can be tailored to different cultures while maintaining fidelity.

In sum, individuals with justice involvement, when compared to the general population, present with unique treatment needs that must be considered when preparing DTx and digital health interventions. These needs are differentiated and shaped by life course experiences and social identity characteristics, such as cultural/racial/ethnic identity, gender identity, adverse childhood experiences, SUD type and severity, and negative experiences with the healthcare and/or justice system(s), which can engender feelings of distrust and/or stigma. Although the criminal justice system was an early adopter of digital health initiatives, it has not remained at the forefront of innovation for those with SUD.

It is critical that technological advancements be available to justice-involved populations if the digital healthcare gap in correctional settings is to be closed. State and federal prisons need to have the infrastructure necessary to support digital health services, including DTx, while assuring that security and privacy issues are addressed and maintained.

## Conclusions

We investigated DTx as a treatment for SUD, reviewing the use and implementation of digital health and DTx for SUD treatment in criminal justice settings and exploring the implementation of DTx for SUD treatment both generally and within criminal justice settings.

### DTx for SUD

Much research indicates that digital health and DTx assist individuals with SUD, treating those with nicotine/tobacco, alcohol, cocaine, cannabis, and opioids [44]. However, when looking at FDA-approved DTx interventions for SUD, only three options exist: reSET, reSET-O, and DynamiCare Health. Only one, TES, has been studied in criminal justice settings where it was found to be equally effective compared to standard treatment in reducing criminality, relapse, and HIV risk behavior [46]. Despite Pear Therapeutics’ recent bankruptcy filing, the evidence base for reSET and reSET-O is strong. DynamiCare Health is approved for smoking cessation during pregnancy, but evidence related to other SUD types is emerging [61, 62]. Five additional DTx interventions were identified; each has been studied and found to be at least somewhat successful. The evidence for CBT4CBT dates back to 2008 [67••]; it has been used with multiple populations [71, 72••] and SUD types [68, 69], and it has been studied in both clinical [67••] and real-world settings [73•]. The evidence for A-CHESS is moderate, with studies demonstrating success in treatment for SUD [75] and less risk of relapse [76]. It has been studied in multiple populations [76, 77] including justice-involved individuals [34]. The other three emerging DTxs, Clickotine, We The Village, and Laddr, are promising but need additional research with other populations and SUDs in order to demonstrate their effectiveness.

### Questions regarding DTx

We also must consider questions raised about digital health and DTx. The most often mentioned are below.

1. Do digital health/DTxs engage clients long enough for their condition(s) to improve [105]? Studies involving reSet, reSet-O, and CBT4CBT have demonstrated increased adherence to SUD treatment (e.g., 17, 22, 64, 68, 73•).
2. Are staff and administrators trained appropriately to use the technology [106]? This question is site-specific. In some cases, there may be issues with the cost and time to train staff and administrators. However, at least one study identified indicated that staff had been trained and were able to assist study participants with issues related to the technology [34].
3. Can the time to complete the regulatory requirements for new DTx be lessened [107, 108]? This issue is unknown. The FDA can change the regulatory requirements but has not yet done so regarding digital health/DTx.
4. Will insurance and/or Medicare approve the use of digital health modalities [104, 105]? This gradually is becoming less of an issue. Specific to the question, Florida has approved Medicaid payments for DTx [99]. Another study found that the use of reSET among individuals with SUD was associated with significant reductions in healthcare resource utilization and costs and concluded that reSET provided an “economic benefit” [15]. A final study found that some insurance companies do cover reSET-O and A-CHESS [66].

### DTx in Criminal Justice Settings

In spite of the number of studies focusing on treatments for SUD, few have been completed in criminal justice settings. Challenges to DTx in such settings can be the perceived expense of the products, lack of staffing within the carceral setting dampening interest in novel approaches, data collection difficulties, privacy fears and distrust of the system on the part of potential participants, and concern about continuing treatment upon reentry with family and friends. However, concerns can be overcome, and some (e.g., cost of DTxs [24, 25, 53–57]) have been shown to be false.

The most basic conclusion is that the justice-involved population, those in carceral settings and on probation or parole, are among the least likely to receive DTx for SUD. Given the proportion of these individuals who are estimated to enter a carceral setting with an SUD, the lack of research in the area and the concomitant lack of DTx must be corrected. This is especially important given that DTx, because they can be delivered both within and outside the carceral setting, may reduce the stigma associated with going to clinics known to treat stigmatized disorders (e.g., 66).

To make such improvements, researchers, leaders, and carceral staff must access evidence-based treatments (e.g., DTx) and remove existing barriers to treatment. Specifically, we recommend

- Addressing gaps in the digital and technological infrastructure of jails and prisons that inhibit access to digital healthcare and therapeutics;
- Decreasing regulatory requirements for DTx that utilize evidence-based treatments (e.g., CBT, TES);
- Defining a Medicare/Medicaid coverage pathway and reimbursement framework for DTx;
- Increasing funding for longitudinal research to evaluate the effectiveness of DTx; and
- Establishing partnerships between DTx companies and Departments of Corrections to streamline access to DTx treatments for individuals with SUD in carceral settings.

## Figures and Tables

**Table 1:** Summary of SUD digital therapeutics (DTx) by SUD type, target population, and FDA approval status

DTx	SUD type	Target population	Device classification	EBI/theory	Company	FDA approval status	Effectiveness
reSET	SUD	Individuals with SUD, justice-involved	Mobile app	CBT, CRA	Pear Therapeutics	FDA-de novo	Strong***
reSET-O	OUD	Individuals with OUD	Mobile app	CBT, CRA	Pear Therapeutics	FDA-510(k)	Strong***
DynamiCare	Smoking cessation	Pregnant women	Mobile app	Contingency management	DynamiCare Health	FDA Breakthrough Device	Moderate**
CBT4CBT	AUD, cocaine use disorder, OUD	Individuals with SUD, Spanish-speaking	Web-based intervention	CBT	CBT4CBT, LLC	–	Strong***
A-CHESS	AUD, SUD	Individuals with AUD and/or SUD	Mobile app	A-CHESS	CHESS Health	–	Moderate**
Clickotine	Smoking cessation	Individuals with tobacco use disorder	Mobile app	US clinical practice guidelines for smoking cessation	Click Therapeutics	–	Promising*
We the Village	AUD	Significant others of problem drinkers	Web-based Intervention	CRA & family training	We the Village, Inc	–	Promising*
Laddr	Problematic alcohol use	Spanish-speaking individuals	Mobile app	CRA	Square2 Systems, Inc	–	Promising*

FDA-510(k): premarket notification and class 1 and 2 medical devices are applicable; FDA-de novo: stands for Evaluation of Automatic Class III Designation and is a special expedited permission system that is given when the safety of a new concept digital healthcare technology that does not have a product classification is proven to be at a certain level; FDA Breakthrough: the device provides for more effective treatment or diagnosis of life-threatening or irreversibly debilitating human disease or conditions and represents Breakthrough Technology, No Approved or Cleared Alternatives Exist, Offers Significant Advantages over Existing Approved or Cleared Alternatives, OR Device Availability is in the Best Interest of Patients, qualifies for all Fast Track designation features

*DTx* digital therapeutics, *SUD* substance use disorder, *EBI* evidence-based intervention, *FDA* Food and Drug Administration, *CBT* cognitive behavioral therapy, *CRA* community reinforcement approach, *OUD* opioid use disorder, *AUD* alcohol use disorder

* Indicates DTx has a limited evidence base with few to no studies assessing real-world applications or increased access to different populations and SUD types

** Indicates DTx has a strong evidence base (multiple RCTs assessing efficacy and durability) and is well validated in both clinical and real-world settings but may have few studies examining application across multiple populations and/or SUD types

*** Indicates DTx has a strong evidence base (multiple RCTs assessing efficacy and durability) and is well validated in both clinical and real-world settings, across multiple populations (e.g., women, non-English speaking) and SUD types
